# Study on Dynamic and Static Mechanical Properties of Copper-Plated Steel-Fiber-Reinforced Self-Compacting Concrete

**DOI:** 10.3390/ma16114025

**Published:** 2023-05-28

**Authors:** Juan Qi, Aonan Liu, Peng Su, Chaomin Mu

**Affiliations:** 1 School of Electrical and Information Engineering, Anhui University of Science and Technology, Huainan 232001, China; 2 School of Safety Science and Engineering, Anhui University of Science and Technology, Huainan 232001, China; 3 Yankuang Energy Group Company Co., Ltd., Jining 272169, China; 4 School of Civil Engineering and Architecture, Anhui University of Science and Technology, Huainan 232001, China

**Keywords:** SCC, CPSF, SHPB, mechanical properties

## Abstract

The mechanical properties and impact resistance of conventional self-compacting concrete (SCC) need to be further improved. In order to explore the dynamic and static mechanical properties of copper-plated steel-fiber-reinforced self-compacting concrete (CPSFRSCC), the static mechanical properties and dynamic mechanical properties of CPSFRSCC with a different volume fraction of copper-plated steel fiber (CPSF) are tested, and a numerical experiment is carried out to analyze the experimental results. The results show that the mechanical properties of self-compacting concrete (SCC) can be effectively improved by adding CPSF, especially for the tensile mechanical properties. The static tensile strength of CPSFRSCC shows a trend that increases with the increase in the volume fraction of CPSF and then reaches the maximum when the volume fraction of CPSF is 3%. The dynamic tensile strength of CPSFRSCC shows a trend that increases first and then decrease with the increase in the volume fraction of CPSF, and then reaches the maximum when the volume fraction of CPSF is 2%. The results of the numerical simulation show that the failure morphology of CPSFRSCC is closely related to the content of CPSF; with the increase in the volume fraction of CPSF, the fracture morphology of the specimen gradually evolves from complete fracture to incomplete fracture.

## 1. Introduction

Self-compacting concrete (SCC), a special kind of concrete, is widely used in construction engineering, underground space engineering, and other fields because it does not need artificial vibration and can rely on its own gravity to fill density. It also has strong fluidity and filling, as well as strong segregation resistance and water resistance [1,2,3]. However, SCC is a typical brittle material, specifically a type of concrete, with the characteristics of compressive strength but poor tensile strength [1,4,5]. In addition, some engineering structures using SCC inevitably face the threat of dynamic loads, such as explosions. The dynamic mechanical properties of engineering materials are received the same attention as the static mechanical properties in the contemporary engineering field [6,7]. However, compared with ordinary concrete materials, the impact resistance of SCC needs to be improved [8,9]. Therefore, it is necessary to develop a new type of concrete, which has the advantages of SCC and strong impact resistance.

Research shows that an effective method to improve the performance of concrete is the addition of fiber materials into concrete [10,11,12,13], such as glass fiber [14], polypropylene fiber [15], carbon fiber [16], and steel fiber [17]. Limited by cost performance and fiber properties [18,19], some kinds of fibers are difficult to use widely in the large-scale production of building materials. Steel fiber, with advantages of low price, high strength, and high temperature resistance [17], is an ideal choice as modified fiber applied in concrete. Researchers have carried out different aspects of research on steel-fiber-reinforced concrete and steel fiber self-compacting concrete, such as electrical conductivity, mechanical properties, permeability, and corrosion resistance [20,21,22]. A large number of mechanical experiments have been applied to the study of the mechanical properties of steel-fiber-reinforced concrete (SFRC) and steel-fiber-reinforced self-compacting concrete (SFRSCC) [23,24,25] because mechanical properties are the most important indicators of the performance of concrete [26,27]. Some scholars have noticed that there is a certain relationship between the mechanical properties of SFRC and the added steel fiber: although steel fibers can improve the mechanical properties of concrete within a certain fiber content, when the content of steel fiber exceeds a threshold, steel fiber is prone to agglomeration, resulting in a decline in concrete performance [28]. This phenomenon seems not to be an example, and it is more obvious in steel fiber self-compacting concrete. Li et al. [29] found that when the content of steel fiber is 1%, steel fiber can better improve the mechanical properties of SCC in the experiment using the split Hopkinson pressure bar (SHPB) to study the dynamic and static compression properties of SCC (0.5%, 0.75% and 1.0%) with different contents of steel fiber. Liao et al. [30] found that the effect of fiber content on the strength of SFRC is more obvious with the increase in strain rate; the compressive strength is the highest when the fiber content is 2.0%, but when the fiber volume fraction exceeds 2.0%, the dynamic compressive strength decreases. Zhao et al. [31] found that with the increase in the volume fraction of steel fiber, the compressive strain at the peak stress of the stress–strain curve increases, and the slope of the decreasing part decreases. In the study of fiber-reinforced recycled-aggregate self-compacting concrete, Aslani et al. [32] found that when the amount of steel fiber substitution reached 0.75%, the strength of the specimen was better promoted. Some scholars have tried to use modified steel fiber instead of traditional steel fiber to improve this problem [33,34]. Among these modified steel fiber materials, copper-plated steel fiber (CPSF) has better hydrophilicity than traditional steel fiber because its surface can better adhere to free water, easily combine with concrete slurry, and does not easily form fiber clusters; consequently, CPSF is attracting the attention of researchers [35].

To sum up, although researchers are carrying out research on how CPSF improves the performance of concrete, reports on how CPSF affects the performance of SCC with CPSF are rare. In addition, how to improve the mechanical properties of copper-plated steel-fiber-reinforced concrete (CPSFRCC) according to the characteristics of CPSF to improve the performance of CPSFRCC under impact loading is also worth further exploration. In this paper, in order to explore the influence of the volume fraction of CPSF on the dynamic and static mechanical properties of CPSFRCC, CPSFRCC specimens with various volume fraction of CPSF were configured separately. A series of dynamic and static mechanical experiments were applied to the dynamic and static mechanical properties test of CPSFRCC specimens with a different volume fraction of CPSF. The effects of volume fraction of CPSF on the static compressive strength, static tensile strength, dynamic tensile strength, dynamic growth factor, and failure mode of CPSFRC are discussed. In addition, the numerical simulation software ABAQUS 2021 was applied to simulate the SHPB experiment of CPSFRC to verify the experimental results. The results provide a theoretical basis for the evaluation of engineering structures using copper-plated steel-fiber-reinforced concrete.

## 2. Materials and Experimental Program

### 2.1. Raw Material

**Figure 1:**
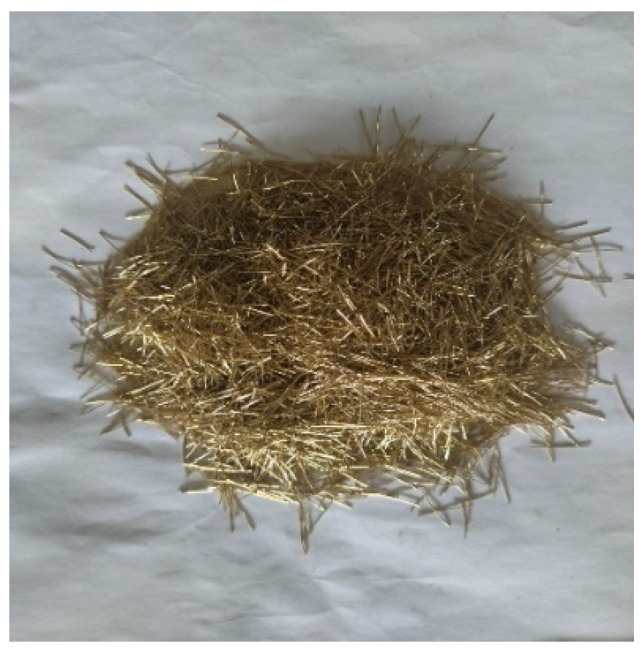
Long straight CPSF in experiment.

CPSFRCC, a high-performance concrete prepared by adding CPSF to the foundation of ordinary SCC, is generally prepared by coarse aggregate, fine aggregate, inorganic cementitious material, high-performance admixture, and water according to appropriate proportions. The raw materials used in the CPSFRCC in the test involved in this paper are as follows: Portland cement, natural river sand, quartz powder, fly ash, water, water reducer, and CPSF. P.052.5 ordinary Portland cement produced by Hejin Yumen was selected, and its performance met the requirements of Chinese standard JGJ/T283-2012. Studies show that the use of some quartz powder instead of natural river sand can effectively improve the fluidity of self-compacting concrete slurry [36]; therefore, 325-mesh Quartz powder was selected to replace part of the river sand. The average density of fly ash used in this experiment was 2.1 g/cm^3^. The water reducer with a water-reduction rate of ≥45% can be selected. Many types of steel fibers are designed, manufactured, and used in engineering construction. Steel fibers, such as flat, hook, and wave, are commonly used [37]. Long straight CPSF (Figure 1) was used in this experiment with a length of 12 mm and a diameter of 220 μm.

### 2.2. Mix Proportion

**Figure 2:**
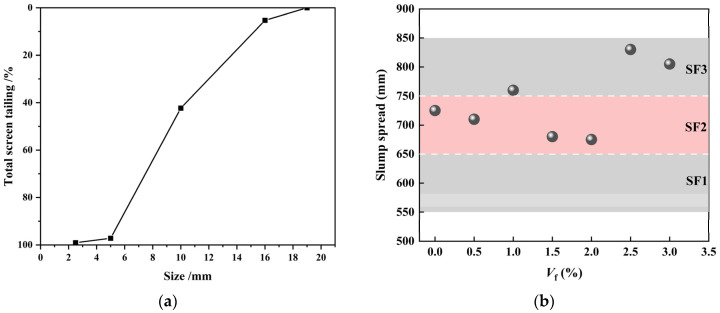
The grading curve of coarse aggregate and slump spread of CPSFRSCC: (**a**) grading curve of coarse aggregate; (**b**) slump spread of CPSFRSCC with different volume fractions of CPSF.

In this experiment, the mix proportion of C60 strength grade CPSFRSCC was determined, as shown in Table 1, according to the Chinese standard JGJ/T283-2012. It should be noted that the SCC was added with CPSF at 7 different volume fractions of 0%, 0.5%, 1%, 1.5%, 2%, 2.5%, and 3%, which were expressed as CPSF0, CPSF0.5, CPSF1, CPSF1.5, CPSF2, CPSF2.5, and CPSF3, respectively. For example, CPSF0 represents SCC without CPSFs, while CPSF1.5 represents SCC with 1.5% volume ratio of CPSF. In order to ensure that the slump of CPSF concrete met the requirements of the Chinese standard JGJ/T283-2012, the slump test was carried out before the pouring of CPSFRSCC with different dosages. The test results are shown in Figure 2b.

### 2.3. Processing of Concrete Specimen

**Figure 3:**
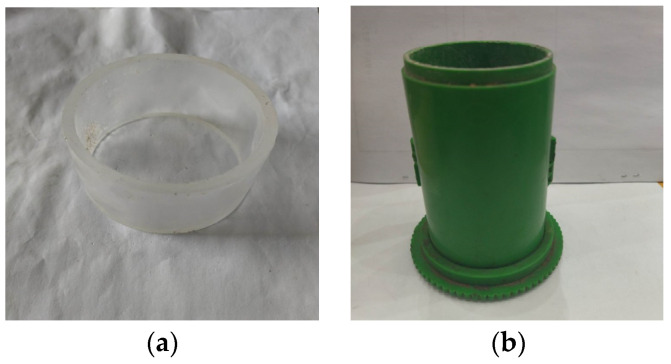
The mold applied in the processing: (**a**) disk specimen mold; (**b**) cylindrical specimen mold.

**Figure 4:**
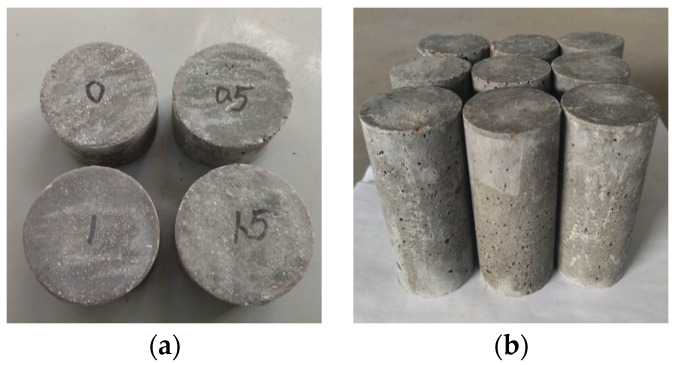
The specimens: (**a**) disk specimens; (**b**) cylindrical specimens.

In order to control variables, all CPSFRSCC specimens underwent the same processing and curing process. In this paper, the production of CPSFRSCC specimens was improved using the production method of ordinary steel fiber self-compacting concrete (SFRSCC) specimens. In order to make the most effective use of CPSF, in the process of making CPSF specimens, the CPSF should be uniformly distributed in the concrete matrix as much as possible. Therefore, the natural river sand, quartz powder, fly ash, cement, and water-reducing agent were mixed and stirred for 3 min to ensure that the concrete had good fluidity. Then, the CPSF was evenly spread into the container, fully stirred for at least 3 min, and finally slowly added. The water was fully stirred for at least 6 min, so that the CPSF could be evenly distributed in the mixing material as much as possible, effectively avoiding the influence of adding water first, which causes the steel fiber to be bound by the cement so it cannot be dispersed. All the specimens experienced the same curing process. After the completion of production, the mold (Figure 3) with slurry was placed in a constant-temperature curing room for 1 day to demold. After demolding, the specimens (Figure 4) with various CPSF contents were marked with pens and were finally placed in a constant-temperature room for 28 days.

### 2.4. Equipment and Principle

#### 2.4.1. SHPB Test

**Figure 5:**
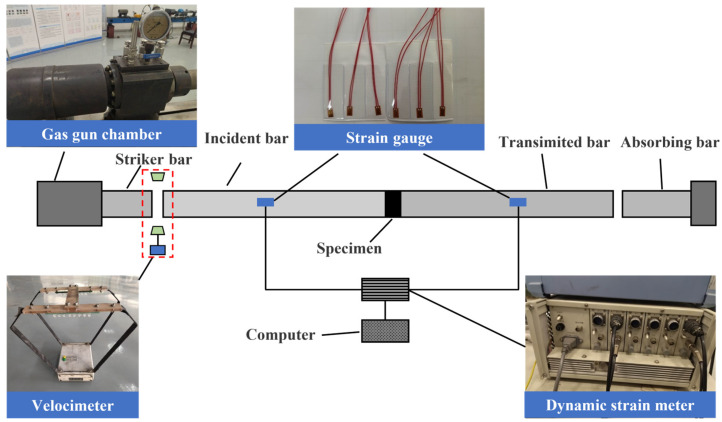
Schematic diagram of the SHPB.

The split Hopkinson pressure bar (SHPB) shown in Figure 5 was applied for the testing of the dynamic mechanical properties of CPSFRSCC specimens. The SHPB system consists of an incident bar, transmission bar, absorbing bar, striker bar, dynamic strain meter, velocimeter, and data acquisition system. Among them, the striker bar, incident bar, and transmission bar had the same diameter of 75 mm, and the lengths were 0.4 m, 4 m, and 2.5 m, respectively. The material was alloy steel, the density was 7850 Kg/m^3^, the wave velocity was 5190 m/s, and the elastic modulus was 195 GPa.

The SHPB experiment was based on two assumptions: the one-dimensional stress wave assumption and the uniform stress distribution assumption [38,39]. The average strain rate and the strain and stress of the specimen can be calculated based on these two assumptions as shown in Equations (1)–(3).

(1) $$\dot{\varepsilon_{s}}\left(t\right)=\frac{V_{1}-V_{2}}{L_{s}}=\frac{C_{0}}{L_{s}}\left[\varepsilon_{i}\left(t\right)-\varepsilon_{r}\left(t\right)-\varepsilon_{t}\left(t\right)\right],$$

(2) $$\varepsilon_{s}\left(t\right)=\int_{0}^{t}\frac{C_{0}}{L_{s}}\left[\varepsilon_{i}\left(t\right)-\varepsilon_{r}\left(t\right)-\varepsilon_{t}\left(t\right)\right]dt,$$

(3) $$\sigma_{s}\left(t\right)=\frac{F_{1}\left(t\right)+F_{2}\left(t\right)}{2S_{s}}=\frac{S_{B}E}{2S_{s}}\left[\varepsilon_{i}\left(t\right)+\varepsilon_{r}\left(t\right)+\varepsilon_{t}\left(t\right)\right],$$

where $\varepsilon_{i}\left(t\right)$, $\varepsilon_{r}\left(t\right)$, and $\varepsilon_{t}\left(t\right)$ represent the incident strain, reflected strain, and transmitted strain collected by the strain gauge, respectively; *E*, $S_{B}$, and $C_{0}$ represent the elastic modulus, section area, and wave velocity of the steel bar, respectively; $V_{1}\left(t\right)$ and $V_{2}\left(t\right)$ represent the velocity on both sides of the contact between the specimen and the bar; $L_{s}$ and $S_{s}$ represent the length and cross-sectional area of the specimen, respectively; and $\dot{\varepsilon_{s}}\left(t\right)$, $\varepsilon_{s}\left(t\right)$ and $\sigma_{s}\left(t\right)$ represent the average strain rate and the strain and stress of the specimen in the SHPB experiment, respectively.

The Brazilian disk-splitting method is widely used in the tensile properties of brittle materials, such as concrete, due to the difficulty of the directly tensile strength testing of such brittle materials [40,41].The traditional Brazilian disk-splitting test applies disk specimens with the theoretical assumption of the Brazilian disk-splitting test that the section of the disk specimen is subjected to the impact force of the incident rod in the radial direction, and the center of the specimen is first broken [42]. When point loading P is performed on a disk specimen with a diameter of D and a thickness of h, the splitting strength formula can be expressed by Equation (4):

(4) $$\sigma_{t}\left(t\right)=\frac{F_{1}\left(t\right)+F_{2}\left(t\right)}{\pi Dh},$$

where $\sigma_{t}\left(t\right)$ represents the tensile strength; $F_{1}\left(t\right)$, $F_{2}\left(t\right)$ represent the loading load on both sides of the specimen; and D and h represent the diameter and thickness of the specimen, respectively.

#### 2.4.2. Static Mechanical Test

**Figure 6:**
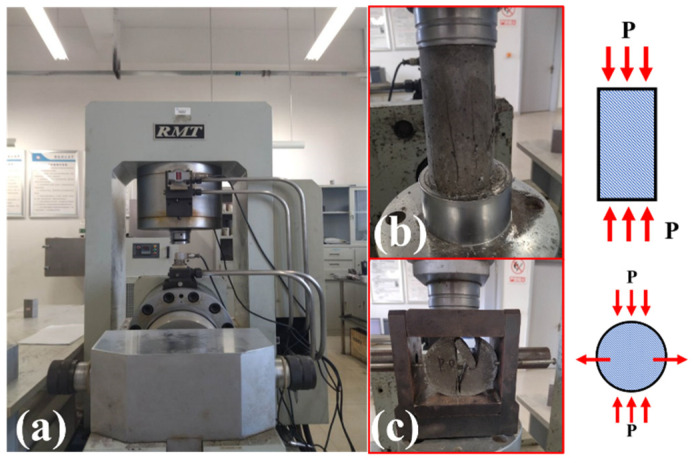
Test equipment and test methods of static mechanical test: (**a**) RMT-150 test system; (**b**) static compression test; (**c**) static Brazilian disk-splitting tensile test.

The static mechanical properties of specimens were tested using an RMT-150 rock mechanical performance test machine (Figure 6a). The RMT-150 rock mechanics test system is mainly used for the mechanical properties test of rock and concrete. It can complete a variety of rock mechanics tests, such as uniaxial compression, uniaxial indirect tension, triaxial compression, and shear [43]. The forms of static compression test and static Brazilian disk-splitting tensile test are shown in Figure 6b,c, and the static compressive strength and static tensile strength of specimen can be obtained through Equations (5) and (6), respectively.

(5) $$\sigma_{d}=\frac{P}{A},$$

where *P* represents the loading load when the specimen fails; and *A* and $\sigma_{d}$ represent the cross-sectional area and compressive strength of the specimen, respectively.

(6) $$\sigma_{t}=\frac{2P}{\pi DL},$$

where *P* represents the loading load when the specimen fails; and *D*, *L*, and $\sigma_{t}$ represent the diameter, thickness, and tensile strength of the specimen, respectively.

## 3. Results and Discussion

### 3.1. Static Mechanical Test

#### 3.1.1. Average Compressive Strength and Tensile Strength of CPSFRSCC with Different Volume Fractions of CPSF

**Figure 7:**
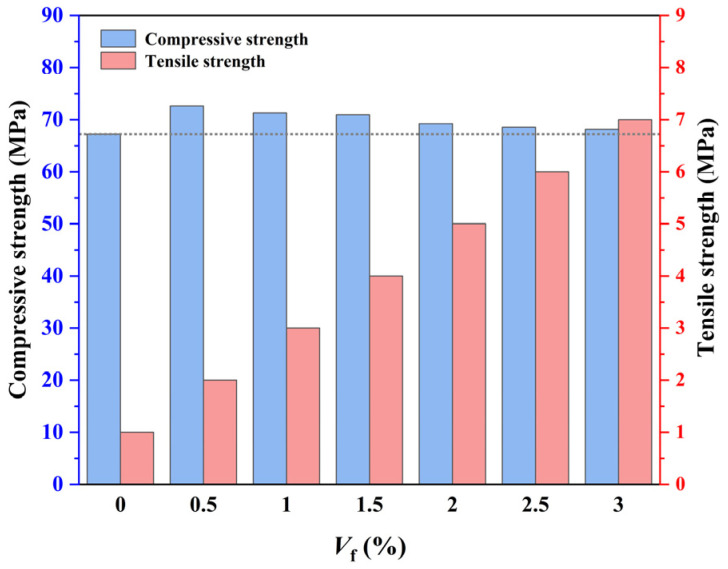
The average compressive strength and tensile strength of CPSFRSCC with different volume fractions of CPSF.

Figure 7 shows the average compressive strength and tensile strength of CPSFRSCC with different volume fractions of CPSF.

It can be observed from Figure 7 that the compressive strength of C60 CPSFRSCC is between 67.22 MPa and 72.63 MPa, which meets the requirements of the corresponding strength grade. The static compressive strength of CPSFRSCC is higher than that without CPSF. The static compressive strength of concrete specimens increased by 8%, 6%, 5.5%, 2.9%, 1.9%, and 1.4% by adding different proportions of CPSF. With the increase in the volume fraction of CPSF from 0.5% to 3%, the compressive strength of CPSFRSCC decreases gradually, reaching the highest value when the volume fraction is 0.5%. The static compressive strength of CPSFRSCC in the 0~0.5% stage increases the most, and the enhancement effect is the most obvious. With the increase in the volume fraction of CPSF, the increase in CPSFRSCC compressive strength is not obvious, and the downward trend begins to appear when the volume fraction of CPSF exceeds 0.5%. The reason for this situation may be attributed to the fact that the volume fraction of CPSF inside the specimen further reduces the inhomogeneity of the matrix of the specimen due to the fluidity of CPSFRSCC, which decreases with the increase in the volume fraction of CPSF.

Compared with the static compressive strength, the effect of adding CPSF on the splitting tensile strength of SCC is more obvious. The splitting tensile strength of concrete specimens with different volume fractions of CPSF increased by 18.1%, 36.1%, 37.5%, 40.1%, 45.6%, and 47.74% compared with CPRF0, showing a disciplined increased with the increase in the volume fraction of CPSF. The above experimental phenomenon is consistent with the findings of Xu et al. [44] in the experiment that explored the effect of the volume fraction of steel fiber on the mechanical properties of high-performance concrete, which found that the volume fraction of steel fiber has a significant effect on the tensile strength of concrete specimens, but the effect on the compressive strength of concrete specimens is not obvious. Xu et al. [44] pointed out that the main reason for this phenomenon is the decrease in the average spacing between fibers caused by the increase in steel fiber content.

#### 3.1.2. Typical Failure Pattern of CPSFRSCC with Different Volume Fractions of CPSF in Static Compression and Tensile Test

**Figure 8:**
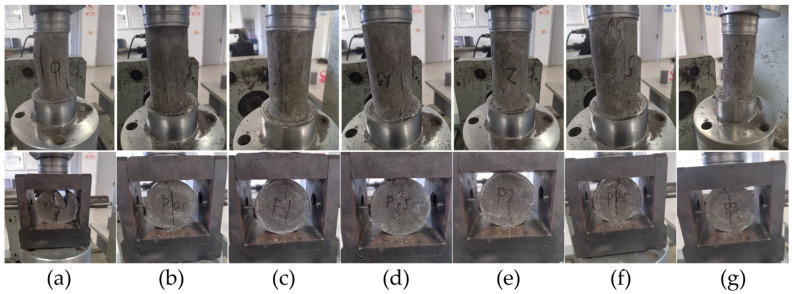
The typical failure pattern of CPSFRSCC with different volume fractions of CPSF in quasi-static compression and tensile test: (**a**) CPSF0; (**b**) CPSF0.5; (**c**) CPSF 1.0; (**d**) CPSF 1.5; (**e**) CPSF 2.0; (**f**) CPSF 2.5; (**g**) CPSF 3.0.

Figure 8 shows the typical failure pattern of CPSFRSCC with different volume fractions of CPSF in the static compression and tensile test. It can be observed from Figure 8 that in the static compression experiment, CPSF0 contains a large number of long and wide cracks after being subjected to static load, and the specimen is almost completely broken. However, there are only small cracks on the surface of SCC mixed with CPSF, and CPSF0 is completely crushed and damaged, which can maintain its original shape and still have certain bearing capacity. In the splitting tensile test, CPSF0 is directly broken into two halves from the middle, and the brittleness characteristics are obvious. The SCC with CPSF is not cracked into two halves, and only cracks are generated at the center line of the disk specimen. In addition, in the splitting tensile test, the cracks of the specimens containing CPSF are reduced, and the fragments after failure are also significantly reduced. The addition of CPSF enhances the friction between the internal matrix of the specimen and reduces the degree of fragmentation of the specimen after tensile failure, which can also prove that the addition of CPSF improves the tensile resistance of concrete specimens. This is consistent with the influence of the volume fraction of CPSF on the static compressive strength and splitting tensile strength of concrete specimens discussed in Section 3.1.1.

### 3.2. Dynamic Tensile Experimental of CPSFRSC

#### 3.2.1. Stress–Strain Curves

**Figure 9:**
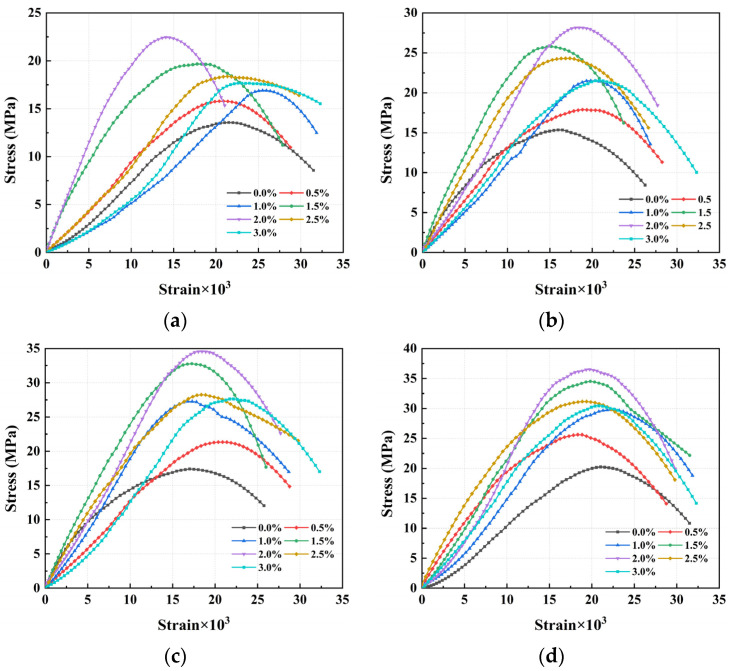
The stress–strain curves of CPSFRSCC with different volume fractions of CPSF under various strain rates: (**a**) 135.33 s^−1^~147.3 s^−1^; (**b**) 200.48 s^−1^~216.46 s^−1^; (**c**) 247.26 s^−1^~268.48 s^−1^; (**d**) 301.38 s^−1^~316.32 s^−1^.

The stress–strain curves of CPSFRSC with different volume fractions of CPSF under virous average impact velocity can be obtained by processing the strain signals obtained in the experiment according to the method described in Section 2, as shown in Figure 9.

It can be seen from Figure 9 that the stress–strain curves of CPSFRSCC can be divided into four stages, showing the similar trend in dynamic tensile stress–strain curves of typical concrete materials [40].

- Initial stage: In the initial stage of dynamic loading, the gap between the internal pores and the aggregate of the specimen is closed due to the loading of the stress wave transmitted along the direction of the bar, showing a slow strain hardening growth stage from the curve.
- Elastic stage: The elastic stage of curve occurs after the initial stage. Similar to other concrete materials, in this stage, the curve shows an approximately linear growth relationship with the accumulation of strain. Some studies show that concrete materials usually reach the elastic stage limit when stress reaches 75% of the peak stress [45], which also applies to the curve of CPSFRSCC.
- Yield stage: With the continuous loading of impact loading and the accumulation of strain, the curve shows a trend in strain softening behavior and a nonlinear growth relationship with the accumulation of strain. The gap between the internal apertures and the aggregate begins to develop continuously, and the specimen shows the characteristics of plastic deformation.
- Failure stage: With the continuous development of the apertures in the specimen and the gap between the aggregates, different degrees of cracks and fracture surfaces are produced. With the further loading of impact loading, the continuous development and interconnection of cracks and fracture surfaces lead to the fragmentation of the specimen, and stress reaches the peak on the curve. However, the specimen after failure still has a certain bearing capacity in a short time, which can be seen from the slow decline after the curve reaches the peak rather than the cliff-like decline.

#### 3.2.2. Dynamic Tensile Strength and DIF of CPSFRSC

**Figure 10:**
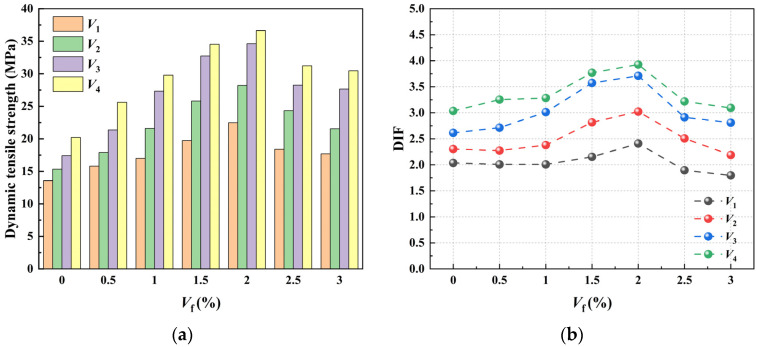
The dynamic tensile strength and DIF of CPSFRSC with different volume fractions of CPSF under various degrees of strain rate: (**a**) Dynamic tensile strength; (**b**) *DIF*.

The average dynamic tensile strength of CPSFRSCC under different loading strain rates is shown in Figure 10a. From Figure 10, it can be observed that at the same strain rate level, the dynamic tensile strength of CPSFRCC shows a trend that increases first and then decreases with the increase in the volume fraction of CPSF. When the volume fraction of CPSF is 2%, the dynamic tensile strength of CPSFRCC reaches its highest level. The dynamic tensile strength of CPSF2 is 65.7%, 83.8%, 98.8%, and 81.2% higher than that of CPSF0 among the four strain rate degrees. In addition to the volume fraction of CPSF, the dynamic tensile strength of CPSFRSCC is also affected by impact loading. Some of the literature uses the concept of the dynamic increase factor (*DIF*, Equation (7)) to investigate the strain rate effect of concrete materials under dynamic loading [8]. The *DIF* of CPSFRSC with different volume fractions of CPSF under various degrees of strain rate is calculated and statistically shown in Figure 10b.

(7) $$DIF=\frac{\sigma_{T,d}}{\sigma_{T,s}},$$

where DIF represents the dynamic increase factor; and $\sigma_{T,d}$ and $\sigma_{T,s}$ represent the dynamic and static strength of the specimen, respectively.

It can be observed from Figure 10b that with the increase in the volume fraction of CPSF, the *DIF* of CPSFRSCC specimens under various loading strain rates shows the same trend. The *DIF* of CPSF0.5 and CPSF1 fluctuates near the initial value and even shows a slight downward trend. When the volume fraction of CPSF reaches 1.5% and 2.0%, the DIF of CPSFRSCC specimens is significantly enhanced and reaches the maximum value when the volume fraction is 2.0%. However, the *DIF* of CPSFRSCC specimens does not increase continuously with the increase in volume fraction. When the volume fraction is 2.5% and 3.0%, it begins to decrease, and the *DIF* value of CPSFRSCC specimens is lower than that of CPSF0.

The above phenomenon shows that CPSF has a significant effect on the dynamic tensile strength of CPSFRSCC. When the concrete matrix is subjected to tensile stress cracking, fine cracks are first generated, then the cracks gradually develop and concentrate; finally, macroscopic cracks are formed, which eventually lead to the fracture of the concrete specimen. CPSF is distributed randomly in the specimen and combines with the concrete matrix to form a fiber–concrete composite. When CPSFRSCC is subjected to tensile loading, the fine cracks encounter steel fibers during the development process, the steel fibers are pulled out from the concrete matrix, and the process of being pulled out consumes additional energy, which makes CPSFRSCC have better tensile performance than conventional concrete.

In summary, the addition of CPSF delays the development of microcracks and the transformation of microcracks to macrocracks by consuming additional energy. Therefore, CPSFRSCC shows the trend that tensile strength increases with the increase in the volume fraction of CPSF. When the volume fraction of CPSF exceeds 2%, the dynamic tensile strength of CPSFRSCC decreases, which may be due to the uneven distribution of a large amount of CPSFs in the SCC matrix. The fiber produces agglomerates, which makes CPSF form a non-uniform complex and leads to the formation of a new weak area bearing external force inside the specimen, resulting in the dynamic tensile strength of CPSFRSCC decreasing instead of increasing.

#### 3.2.3. Failure Process

**Figure 11:**
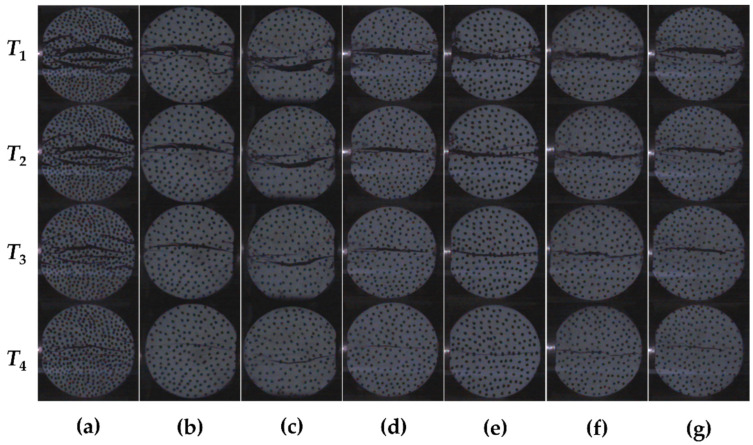
The failure process of CPSFRSCC with different volume fractions of CPSF in SHPB experiment (Strain rate = 135.53 s^−1^~147.3 s^−1^): (**a**) CPSF0; (**b**) CPSF0.5; (**c**) CPSF 1; (**d**) CPSF 1.5; (**e**) CPSF 2; (**f**) CPSF 2.5; (**g**) CPSF 3.

In the SHPB dynamic splitting test, the whole process of CPSFRSCC specimen failure is photographed by a high-speed camera. Four failure images of CPSFRSCC at different times are selected in turn, and the failure process of seven different CPSF volume fractions are analyzed. The dynamic splitting failure process of the CPSFRSCC specimen under a loading air pressure of 0.2 MPa (strain rate range from approximately 135.53 s^−1^ to 147.3 s^−1^) is shown in Figure 11.

Through Figure 11, it can be observed that the cracks of CPSF0 (Figure 11a) appear at the center line of the specimen, then the numbers of parallel cracks begin to appear near the center line, finally breaking into numbers of fragments, showing obvious brittle failure characteristics. However, the situation for CPSFRSCC (CPSF0.5-CPSF3, Figure 11b–g) is not the same. The cracks of CPSFRSCC at the center line are significantly reduced, and the number of cracks decrease with the increase in volume fraction. The specimen only produces one main crack, and the specimen is basically evenly broken into two pieces. Additionally, the specimen begins to break from the center of the specimen and the contact surface between the specimen and the bar when the volume fraction of CPSF exceeds 1%. In addition, the crack initiation mode of CPSFRSCC is the same as that of conventional SCC (CPSF0), which is extended from the center to the edge of the specimen.

### 3.3. Numerical Simulation of Dynamic Failure Characteristics of CPSFRSC

#### 3.3.1. Modeling

**Figure 12:**
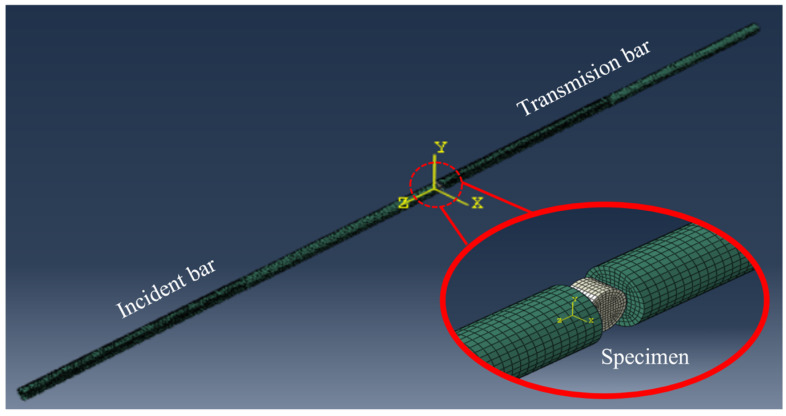
Finite element model of SHPB.

In order to verify the SHPB experiment, the numerical simulation software ABAQUS is applied to simulate the SHPB experiment of CPSFRSCC. The finite element model is established in the software as shown in Figure 12, consisting of a striker bar, incident bar, the CPSFRSCC specimen, and a transmission bar. The end diameter of the steel bar is 75 mm, and the material is alloy steel. The end diameter of the specimen is 65 mm, and the thickness *D* is 35 mm. The material parameters of the steel bar are shown in Table 2.

#### 3.3.2. Constitutive Model and Material Parameters

The CDP model is selected as the constitutive model of the CPSFRSCC specimen in this simulation according to the method given in the literature [46]. The model parameters of concrete in this simulation are shown in Table 3. Fang et al.’s [47,48] study shows that ABAQUS needs to set the relationship between the expansion angle and the stress–strain relationship when using the damage plasticity model. In the literature [49], the expansion angle is set to 35°, and the ratio of stress to strain is usually selected as 1.160; meanwhile, the default value of eccentricity is 0.1 and *K*_c_ is generally 0.6667. The value of the viscosity parameter should be appropriate, and the value is selected as 0.001 in this simulation. The Johnson–Cook (JC) model [50] is selected as the material model of CPSF, and the material parameters are showed in Table 4.

#### 3.3.3. Randomly Distributed of Fiber

**Figure 13:**
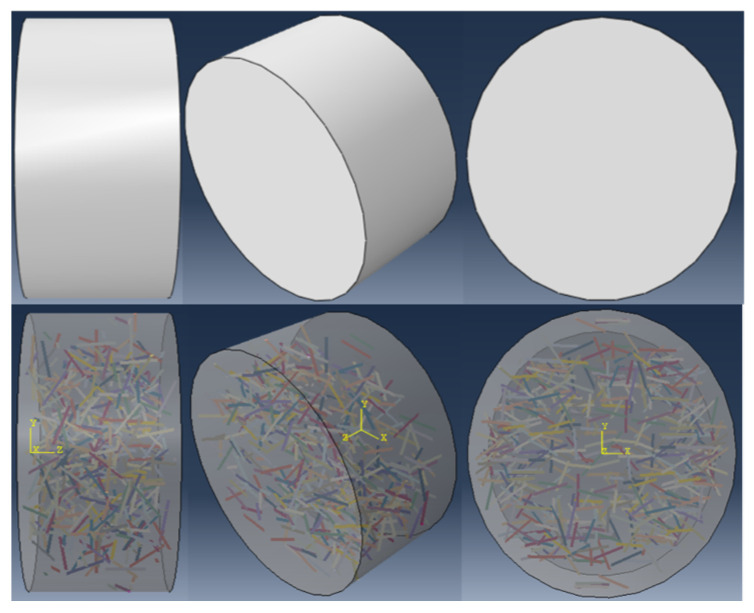
Modeling of CPSFRSS specimen.

**Figure 14:**
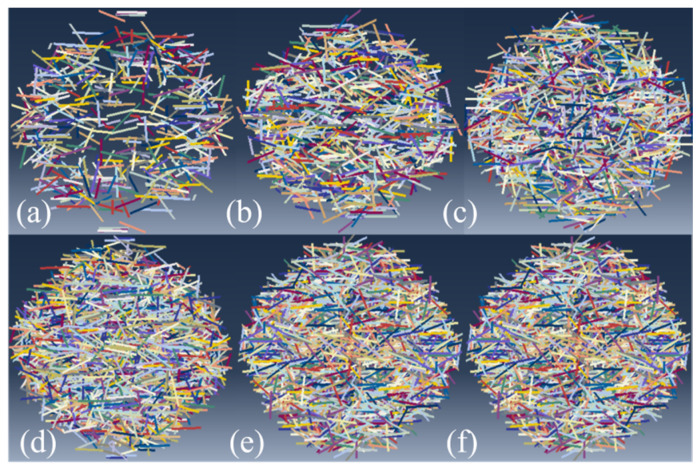
Random distribution of various volume fractions of steel fibers: (**a**) CPSF0.5; (**b**) CPSF1; (**c**) CPSF 1.5; (**d**) CPSF 2; (**e**) CPSF 2.5; (**f**) CPSF 3.

A straight line of 12 mm is randomly generated in a disk model with a diameter of 65 mm and a height of 35 mm (Figure 13) to represent CPSF. The cross-sectional diameter of the linear CPSF is 0.22 mm. The total volumes of all linear CPSFs generated by the plug-in [51] in ABAQUS are 0%, 0.5%, 1%, 1.5%, 2%, 2.5%, and 3% of the volume fraction of the disk specimen model, and the CPSFs are disorderly distributed in the CPSFRSCC disk specimen. The numerical models of the CPSFRSCC specimen with various volume fractions of CPSF can be obtained as shown in Figure 14. In this simulation, the mesh division module in ABAQUS 2021 software is selected, and the SCC matrix and CPSF are quadrilateral mesh. The boundary condition is set to general contact, and the friction is 0.1.

#### 3.3.4. Results and Verification

**Figure 15:**
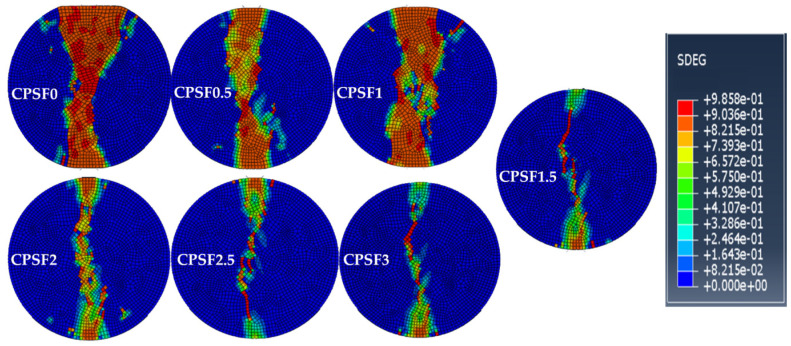
Failure morphology of CPSFRSCC specimens in numerical simulation.

**Figure 16:**
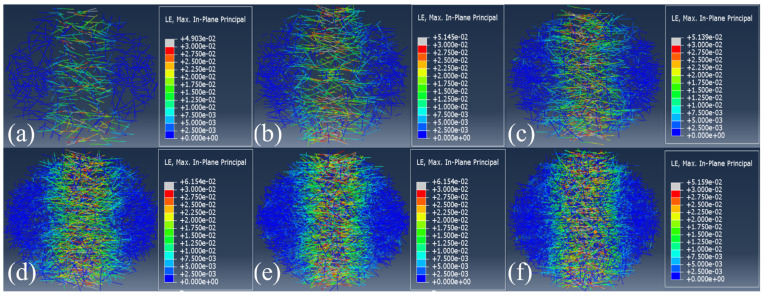
The distribution of CPSF in specimens in numerical simulation: (**a**) CPSF0.5; (**b**) CPSF1; (**c**) CPSF 1.5; (**d**) CPSF 2; (**e**) CPSF 2.5; (**f**) CPSF 3.

Figure 15 and Figure 16 show the final failure morphology of the CPSFRSCC specimens with different volume fractions of CPSF during the whole process of impact loading and the distribution of CPSF in the specimens after impact loading.

It can be observed from Figure 15 and Figure 16 that the failure morphology of the specimens without CPSF and the specimens with CPSF are significantly different. The disk specimen without CPSF (CPSF0) is completely broken with a wide crack, and the width of the two ends of the crack is greater than the width of the crack near the center of the disk specimen. The specimen is evenly divided into two halves, showing obvious brittle failure characteristics. As for CPSF0.5 and CPSF1, the width of the crack of the failure specimen is slightly reduced, and the disk specimen is almost completely broken, but there is little traction near the center of the specimen, and the two sides of the crack of the specimen are evenly divided into two halves. When the volume fraction of CPSF exceeds 1.5, the crack width is obviously reduced, the crack is slightly curved, and the width of both ends of the crack is greater than the width of the crack near the center of the disk specimen. The specimen is not completely broken, and the two sides of the crack are equally divided into two halves. The above simulation results show that compared with the specimens without CPSF (CPSF0), the crack width of the specimens with CPSF is reduced to varying degrees after failure, and the decrease in crack width increases with the increase in the volume fraction of CPSF. In addition, with the increase in the volume fraction of CPSF, the fracture form of the specimen gradually evolves from complete fracture to incomplete fracture.

It seems that there are some differences in the final failure mode of CPSFRSCC in the experiment and numerical simulation. In the SHPB experiment, almost all of the CPSFRSCC specimens are complete failures; only a few specimens with a higher volume fraction of CPSF are not completely broken, while only the specimens without CPSF and the specimens with a lower volume fraction of CPSF are completely broken in the numerical simulation. This phenomenon has also appeared in other studies [52,53]. Chen et al. [54] attributed these reasons to the unevenness of the artificially mixed concrete specimens used in the experiment. The artificially made specimens are inhomogeneous and there are pores in the concrete matrix, or CPSF produces agglomeration, which affects the concrete matrix structure. However, in the numerical simulation, these two cases do not occur. The specimens are homogeneous and can be uniformly stressed. The CPSF in the specimens are randomly distributed, and there is no steel fiber agglomeration. In addition, during the SHPB experiment, due to the difference in the size of the bar and the specimen, it is impossible to ensure that the axial center of the specimen is completely aligned with the center of the compression bar when the CPSFRSCC specimen is placed, which in turn affects the one-dimensional loading of the stress wave [55], resulting in heterogeneous damage to the specimen during the loading process. In general, the results of the numerical simulation are close to that of the experiment, if these error factors are taken into account.

## 4. Conclusions

In order to study the mechanical properties of CPSFRSCC under static and dynamic loads with different volume fractions of CPSF, the static and dynamic mechanical properties of CPSFRSCC are investigated by static mechanical testing, dynamic mechanical testing, and numerical simulation analysis. The conclusions are as follows:

1. The static compressive strength and static tensile strength of CPSFRSCC can be improved by adding CPSF, and the improvement in static tensile strength is more obvious. The tensile strength of CPSFRSCC increases with the increase in the volume fraction of CPSF and reaches its maximum value when the volume fraction of CPSF is 3%.
2. The dynamic tensile strength of CPSFRSCC increases first and then decreases with the increase in the volume fraction of CPSF, reaching its maximum value when the volume fraction of CPSF is 2%. When the volume fraction of CPSF exceeds 2%, the dynamic tensile strength decreased, but the peak tensile strength remains higher than that of conventional SCC. In addition, with the increase in strain rate, CPSFRSCC shows an obvious strain rate effect.
3. The results of numerical simulation show that CPSF has a significant effect on the failure morphology of SCC. The crack width decreases slightly, and the specimen is almost completely broken when 0.5% and 1% of CPSF are mixed; the crack width decreases obviously and the width of both ends of the crack is greater than the width of the crack near the center of the disk specimen, and the specimen is not completely broken when 1.5%, 2%, 2.5%, and 3% of CPSF are mixed.

## Figures and Tables

**Table 1:** The mix proportion of CPSFRSCC (Kg/m^3^).

Types	Cement	Quartz Powder	Sand	Fly Ash	Water Reducer	Water	CPSF
CPSF0	800	260	920	220	16	150	0
CPSF0.5	800	260	920	220	16	150	39
CPSF1	800	260	920	220	16	150	78
CPSF1.5	800	260	920	220	16	150	117
CPSF2	800	260	920	220	16	150	156
CPSF2.5	800	260	920	220	16	150	195
CPSF3	800	260	920	220	16	150	234

**Table 2:** The material parameters of the steel bar.

Density (kg/m^3^)	Elastic Modulus (GPa)	Poisson Ratio
7850	195	0.25

**Table 3:** Concrete model parameters.

Density (Kg/m^3^)	Elastic Modulus (GPa)	Poisson Ratio	*K*_c_	Viscosity	Eccentricity	Expansion Angle (°)
2.366 × 10^3^	33.2	0.2	0.6667	0.001	0.1	35

**Table 4:** The model parameters of CPSF.

Density (g/mm^3^)	Elastic Modulus (GPa)	Poisson Ratio
0.0078	210	0.26

## Data Availability

Not applicable.
